# Targeted effect of ischemic preconditioning on the gas exchange threshold in healthy males and females

**DOI:** 10.1007/s00421-024-05481-8

**Published:** 2024-04-20

**Authors:** M. Goldsmith, J. Siegler, S. Green

**Affiliations:** 1 School of Health Sciences, Western Sydney University, Campbelltown Campus Building 20, Sydney, NSW Australia; 2 College of Health Solutions, Arizona State University, Phoenix, AZ USA

**Keywords:** Ischemic preconditioning, Maximum graded exercise, Cardiac, Ventilatory, Metabolic

## Abstract

Ischemic preconditioning (IPC) appears to improve exercise performance although there is uncertainty about the intensity dependence of this effect. The present study sought to clarify effects of IPC on physiological responses at and below peak oxygen uptake, including the gas exchange threshold (GET). Ten male and female participants completed five cycling ramp tests (10 W/min) to failure, with the final two tests preceded by either IPC (4 × 5 min 220 mmHg bilateral leg occlusions) or SHAM (20 mmHg), in a randomised crossover design. The rates of O_2_ uptake ($\dot{V}$O_2_), carbon dioxide output ($\dot{V}$CO_2_), and expired ventilation ($\dot{V}$_E_) were measured at rest and throughout exercise. Exercise data were fitted using several functions to identify GET, two ventilatory thresholds and peak $\dot{V}$O_2_. IPC increased $\dot{V}$O_2_ at GET by ~ 9% (IPC: 1.89 ± 0.51 L/min, SHAM: 1.73 ± 0.56 L/min; *p* = 0.055) and power output at GET by ~ 11% (IPC: 133 ± 36 W, SHAM: 120 ± 39 W; *p* = 0.022). In addition, peak power output increased by 2.4% following IPC (IPC: 217 ± 50 W, SHAM: 212 ± 51 W; *p* = 0.052), but there was no significant effect of IPC on peak $\dot{V}$O_2_ (IPC: 2.87 ± 0.68 L/min, SHAM: 2.84 ± 0.73 L/min;* p* = 0.60) or the ventilatory thresholds. The present results suggest that IPC improves GET and peak power output but not peak $\dot{V}$O_2_ during a maximal graded test.

**Supplementary Information:**

The online version contains supplementary material available at 10.1007/s00421-024-05481-8.

## Introduction

Ischemic preconditioning (IPC) involves brief episodes of ischemia followed by reperfusion of a target tissue in order to protect it against injury caused by a more prolonged period of ischemia during surgery (Thijssen et al. 2016). Clinical studies reported positive effects of IPC, induced by arterial ligation, on tolerance to a more prolonged period of ischemia during surgery in the heart (Murry et al. 1986), kidneys (Lee and Emala 2000), liver (Yoshizumi et al. 1998) and skeletal muscle (Pang et al. 1995). The IPC procedure was later adapted for studies of human exercise performance, in which brief and intermittent periods of ischemia were induced in lower limbs by the inflating and deflating of limb cuffs prior to exercise. The first of these studies reported a significant improvement in peak power output during an incremental cycling test (de Groot et al. 2010). The recent analysis of outcomes from 52 human studies, however, showed that approximately half of them (*n* = 25) reported significant improvements in exercise performance (Caru et al. 2019).

The physiological mechanisms underlying effects of IPC on human exercise performance are not clear. Insight has been hampered by the use of a wide variety of measurements and many different combinations of exercise mode, test protocol and use of closed- and open-loop exercise tasks, as well as the lack of apparent link between these exercise characteristics and the IPC effect (Caru et al. 2019). A basic problem concerns the uncertain influence of exercise intensity on the IPC effect. In clarifying this, the use of a continuous maximum graded protocol with appropriate measurements is attractive because it enables study of the IPC effect across a wide range of intensity that requires the involvement of all major types of motor units, contributions from aerobic and anaerobic metabolism, and evokes large changes in the cardiorespiratory responses and rate of oxygen uptake ($\dot{V}$O_2_).

Several studies have used maximal graded exercise (treadmill or cycle) to study the physiological effects of IPC (Bailey et al. 2012, Cheung et al. 2020, Clevidence et al. 2012, de Groot et al. 2010, Kaur et al. 2017, Kido et al. 2015, Kilding et al. 2018, Sabino-Carvalho et al. 2017). Peak $\dot{V}$O_2_ was increased by IPC in some (Cruz et al. 2015; de Groot et al. 2010) but not most studies (Cheung et al. 2020, Cocking et al. 2017, Kido et al. 2015, Kilding et al. 2018). There are also conflicting reports about submaximal responses, with a report that IPC delayed the onset of blood lactate accumulation at intensities well below peak $\dot{V}$O_2_ (Bailey et al. 2012) but others which reported no effect of IPC on the lactate threshold (Sabino-Carvalho et al. 2017) or gas exchange threshold (Kilding et al. 2018). Interpretation of this evidence is somewhat undermined by methodological concerns related to precision of measurement of lactate and ventilatory thresholds (see Discussion), especially given that the performance effects of IPC typically observed are small (~ 1–4% improvement).

For this study, we developed an exercise testing and analytical approach which yielded high-precision estimates of the gas exchange threshold (GET) and peak $\dot{V}$O_2_. We investigated effects of IPC on GET and peak $\dot{V}$O_2_, as well as related measurements, following a series of baseline tests. We tested the hypothesis that IPC improves GET and peak $\dot{V}$O_2_.

## Materials and methods

### Participants

Ten (5 female, 5 male) recreationally active participants (age, 31.8 ± 10.5 years; height, 172.9 ± 8.4 cm; weight, 73 ± 9.2 kg) volunteered to take part in the study. Participants were asked to keep a food diary for 24 h prior to testing and replicate it before each trial. Participants were also asked to refrain from alcohol, caffeine, and strenuous exercise for the 24-h period prior to testing. All participants gave written informed consent and completed a physical activity readiness questionnaire (PAR-Q) before any testing took place. Ethical approval was provided by the Western Sydney University Human Research Ethics Committee (H13984).

## Study design

Participants visited the lab on five separate occasions to complete an incremental exercise test on a cycle ergometer (Corival Recumbent, Lode, Groningen, Netherlands) until task failure. The initial two visits were familiarisation sessions with the third visit used as a control condition (CON). The final two tests were preceded by an intervention of either IPC or a sham protocol, in a counterbalanced crossover design. Randomisation was conducted by generating random numbers for each condition for all participants using online software (Urbaniak and Plous 2013). Participants were blinded to the original hypothesis of the study and informed that the aim of the study was to compare the effects of two different cuff pressures of IPC, and that both interventions may improve performance. For each participant, exercise tests were conducted at the same time of day (± 1 h) and experimental trials were separated by a minimum of seven days to ensure no possible carryover effects of IPC (Loukogeorgakis et al. 2005).

## Experimental procedure

On the first visit to the laboratory participants had their height, body mass, and skinfold of the right thigh recorded. Participants were then familiarised with the cycle ergometer before seat height and a self-selected cadence were recorded and replicated for all subsequent trials. Before each test, ambient temperature (°C), relative humidity and barometric pressure (mmHg) were recorded. Participants were then fitted for near-infrared spectroscopy (NIRS) and electrocardiography (ECG) using a 3-lead configuration (lead II). Once seated on the ergometer participants inserted a mouthpiece and attached a nose clip for the collection of expired gas (see Measurements) and remained still and relaxed for 5 min for the collection of resting data. The exercise test began at 0 W and increased by 1 W every 6 s (10 W/min) until task failure. Task failure was reached when cadence dropped by 10 rpm for 5 s or longer and, under control conditions (CON), varied between 1041–1912s (mean ± SD = 1340 ± 236 s).

## IPC/SHAM protocols

**Fig. 1:**
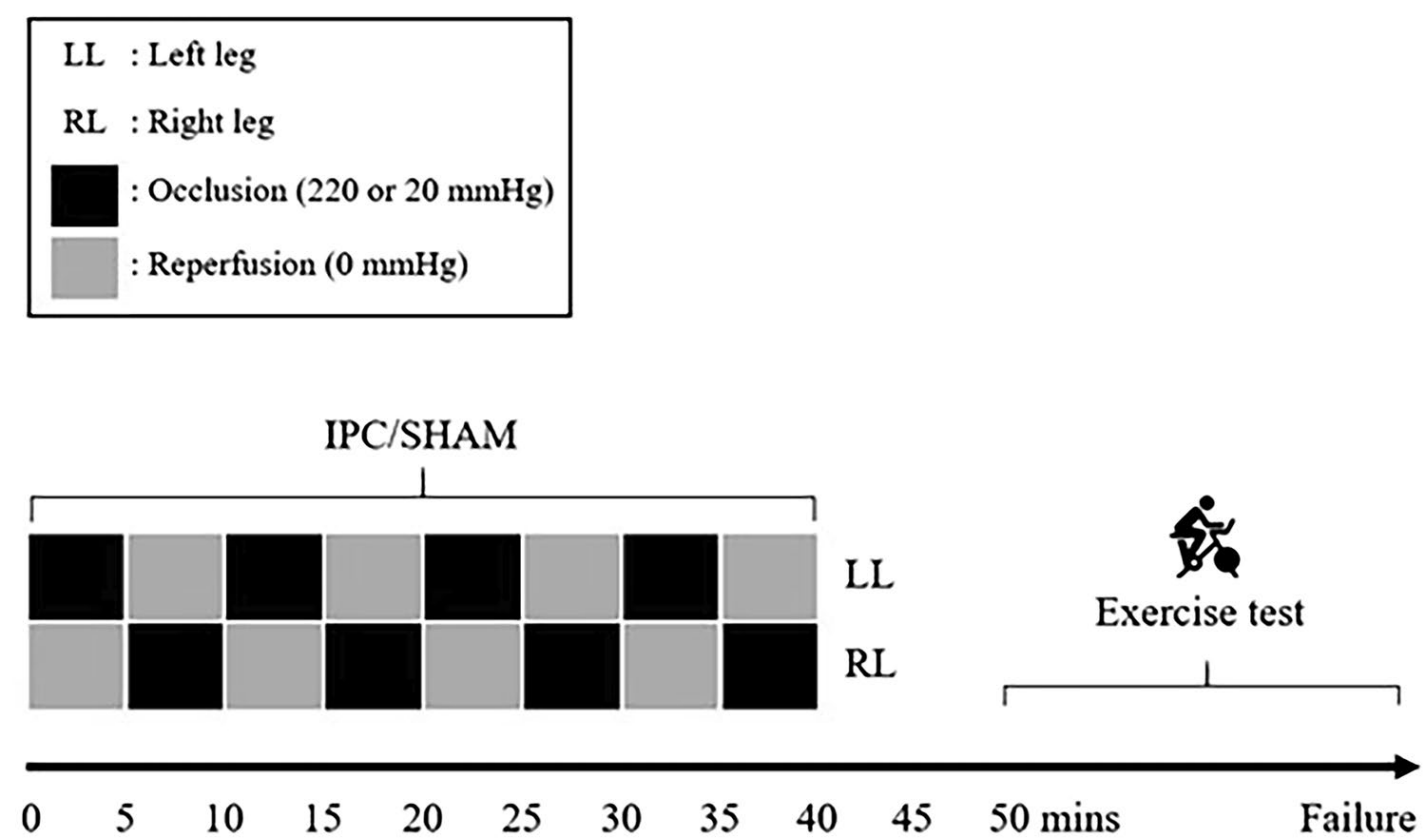
Schematic of ischemic preconditioning (IPC) and sham intervention with protocol timeline

On the fourth and fifth visit an IPC or sham (SHAM) protocol (Fig. 1) was administered before the start of exercise. Both interventions were performed with the participant lying in a supine position with an inflatable tourniquet cuff (4.5" × 34" Easy-FIT Tourniquet Cuff, Delfi Medical Innovations, Vancouver BC, Canada) positioned on each of the upper thighs and pressurised using an automated system (Personalized Tourniquet System, Delfi Medical Innovations, Vancouver BC, Canada). For IPC, ischemia was achieved by inflating thigh cuffs well above the systolic arterial blood pressure to 220 mmHg. For SHAM, thigh cuffs were inflated to 20 mmHg. For IPC and SHAM, both limbs were subjected to four 5-min periods of cuff inflation in an alternating manner and lasting 40 min in total. Participants then waited 10 min following IPC/SHAM before starting measurements at rest (for 5 min) and exercise.

## Measurements

### Pulmonary gas exchange

During the exercise test expired air was measured using an open-circuit system consisting of a 4.7 L mixing chamber (MLA246, ADInstruments, Sydney, NSW, Australia) on the expired side from which gas was continuously sampled and analysed for O_2_ (N22-M, S-3A/I, AEI Technologies, Bastrop TX, USA) and CO_2_ (P-61B, CD-3A, AEI Technologies, Bastrop TX, USA). Gas flow was controlled using a dual channel flow controller (R-2 Flow Control, AEI Technologies, Bastrop TX, USA) and dried using a cartridge containing desiccant and nafion tubing (MLA6024/MLA0343, ADInstruments, Sydney, NSW, Australia). Expired air temperature was measured at the same gas sampling port using a thermistor pod (MLT415, ADInstruments, Sydney, NSW, Australia). Respiratory volumes and frequency were measured using a pneumotachometer (MLT1000L, ADInstruments, Sydney, NSW, Australia) placed at the outlet of the mixing chamber. Analysers were calibrated using the same two known gas concentrations immediately before each test, Nitrogen and Carbogen (16% O_2_, 5% CO_2_). Expired volume was calibrated using a 3 L syringe (Hans Rudolph, Kansas City MO, USA). All outputs were connected to a data acquisition system (PowerLab, ADInstruments, Sydney, NSW, Australia) and recorded using the data analysis software (LabChart7, ADInstruments, Sydney, NSW, Australia).

The data were checked for aberrant breaths (coughing, swallowing, etc.) and removed if they fell by more than 2 standard deviations (SD) below the mean resting value of expired breath volume. Fractions of expired O_2_ (F_E_O_2_) and CO_2_ (F_E_CO_2_) were smoothed using a 12 s average with decimation to remove small respiratory oscillations in these signals. The recordings of F_E_O_2_ and F_E_CO_2_ sampled from the mixing chamber, temperature of expired air in the mixing chamber, as well as the expired volume measured by the pneumotachometer were used for the calculation of $\dot{V}$O_2_ and rates of carbon dioxide output ($\dot{V}$CO_2_), and expired volume ($\dot{V}$_E_). Unpublished evidence from our laboratory showed that the delay in the respiratory gas recordings relative to pneumotachometer, associated with transit of expired air from the mouth to the mixing chamber and the gas stream from this chamber to gas analysers, ranged from ~ 2–10 s during ramp exercise (depending upon $\dot{V}$_E_) but did not significantly influence the fundamental relationships between $\dot{V}$_E_, $\dot{V}$O_2_ and $\dot{V}$CO_2_. The $\dot{V}$O_2_ and $\dot{V}$CO_2_ were calculated using the Haldane transformation and expressed as standard, temperature, pressure, dry (STPD).

### End-tidal gases

In addition to respiratory gases sampled from the mixing chamber (i.e. FeO_2_, FeCO_2_) used in the computation of $\dot{V}$O_2_ and $\dot{V}$CO_2_, respiratory gases were also sampled at the mouth for fractions of O_2_ (FO_2_) and CO_2_ (FCO_2_) using an additional respiratory gas analyser (ML206, ADInstruments, Sydney, NSW, Australia) and recorded at the end of each breath for the calculation of end-tidal FO_2_ and FCO_2_. The behaviours of end-tidal FO_2_ and FCO_2_, rather than FeO_2_ and FeCO_2_ from the mixing chamber, were used to support the estimation of GET (see Respiratory Thresholds below).

### Near-infrared spectroscopy

Oxygenation status of skeletal muscle tissue was assessed using a spatially resolved, dual-wavelength NIRS apparatus (MooreVMS-NIRS, Axminster, Devon, UK). NIRS-derived values for deoxyhemoglobin (HHb) and oxyhemoglobin (O_2_Hb) volumes were assessed in arbitrary units (AU) and the ratio of O_2_Hb to O_2_Hb + HHb used to estimate the tissue saturation index (TSI) expressed as a percentage. The NIRS probe consisted of a detector and an emitter head separated by 30 mm embedded in a black silicon mould placed at the mid-point along the long axis of the vastus lateralis muscle of the right leg. Skinfold thickness was measured at the site of the application of the NIRS using a Harpenden skinfold caliper (British Indicator, Burgess Hill, West Sussex, UK). A skinfold of less than one-half of the distance between emitter and detector was required to ensure adequate light penetration to muscle tissue and data were excluded from the NIRS analysis if not met. The anatomical positioning of optodes was noted during the initial trial and replicated for all subsequent visits. A modified form of the Beer − Lambert law, using two continuous wavelengths (750 and 850 nm) and a differential optical path length factor of 4.95, was used to calculate micromolar changes in tissue haemoglobin concentrations.

## Data analysis

To estimate the various thresholds and peak $\dot{V}$O_2_ ($\dot{V}$O_2peak_), exercise data were fitted to functions as described Table S1. The data recorded during rest prior to exercise were not used in these analyses. To reduce fitting bias induced by the progressive increase in data density with test time, all test data were weighted based on the breathing period of each breath relative to the highest inter-breath period. Fitting was performed using a non-linear least-squares (Marquardt − Levenberg) procedure with convergence of the R^2^ value to six significant digits (TableCurve 2D, Systat Inc.). Fitting was always performed in two stages, with outliers (95% prediction intervals) eliminated after the first stage. All data sets were de-identified before fitting.

### Respiratory thresholds

A fixed sequence of stages of analysis of relationships between variables was followed and details about the various functions used are provided in Table S1. The first and second ventilatory thresholds (VT_1_, VT_2_) were estimated on the basis of the $\dot{V}$O_2_-$\dot{V}$_E_ relationship using a function which accounted for an initial adaptive phase, followed by three linear phases separated by two thresholds. The VT_2_ was taken to represent the respiratory compensation threshold (RCT). Second, GET was estimated using the $\dot{V}$O_2_ − $\dot{V}$CO_2_ relationship by first eliminating all data above RCT, fitting a three-phase function to identify and then eliminate initial data, then fitting a two-phase function to identify the delay of the second phase (in units of $\dot{V}$O_2_) which represents GET. This is a similar process to that described by Beaver et al. (1986), but differs in that these authors arbitrarily eliminated the first minute of test data whereas the present approach formally identified all initial data with a lower slope than subsequent data using a curve-fitting approach. The two-step procedure we developed to identify GET is described in Table S1, with the first step effectively identifying an ‘initial adaptive phase’ of $\dot{V}$CO_2_ with respect to $\dot{V}$O_2_, attributed to changes in body CO_2_ stores by Beaver et al. (1986), using regression analysis as opposed to the arbitrary deletion of initial data (first minute) described by these investigators. Third, the power output at GET was estimated using the $\dot{V}$O_2_ at GET and the $\dot{V}$O_2_-power relationship. Fourth, in the original method the behaviours of end-tidal respiratory gases were used to confirm GET detection (Beaver et al. 1986). In the present study, the timings of the rise in end-tidal FO_2_ and slightly delayed fall in end-tidal FCO_2_, after both of these responses had usually stabilised below GET, were identified through curve-fitting and their $\dot{V}$O_2_ equivalents identified using the $\dot{V}$O_2_ − time relationship (Table S1). The use of these functions and others described in Table S1 is not based on the work of others but rather represented our attempt to use curve fitting to describe the various behaviours of inter-related responses in a consistent manner.

### Oxygen uptake

The $\dot{V}$O_2peak_ was estimated as the maximum predicted value in the $\dot{V}$O_2_-time series. The function selected to describe this series included a final exponential term which could account for either an exponential increase or decrease (‘plateau’) in $\dot{V}$O_2_ towards the end of the test. Peak $\dot{V}$O_2_ is often estimated as the mean of the last 30 s of recordings during ramp exercise. Averaged values recorded during the final 30 s of all ramp tests (*N* = 30) performed across CON, SHAM and IPC conditions (2.80 ± 0.67 L^.^min^−1^) was significantly lower than  maximum values predicted using the $\dot{V}$O_2_-time series (2.85 ± 0.68 L^.^min^−1^). The slope of the $\dot{V}$O_2−_time relationship between *t* = 0 s and the time corresponding to GET ($\dot{V}$O_2_ slope) was also analysed and used as an estimate of exercise economy.

### Near-infrared spectroscopy

A single function was used to analyse the TSI response as a function of time, but in two stages. First, the entire exercise time-series were fitted, and then a time series restricted to ≤ GET was fitted. The total areas under these two TSI-time series (i.e. TSI-time products) were calculated. To ensure consistency in time comparisons up until the point of GET, the time taken to reach GET during the SHAM trial was used as the reference for each participant.

### Heart rate

To analyse heart rate (HR), the beat-to-beat HR was plotted against time and two parameters were extracted: the slope of the HR-time curve (HR slope) and the peak HR. The HR slope was calculated to assess the rate of change in HR over time. The peak HR was determined as the highest HR reached during the exercise test.

## Statistical analysis

Statistical analysis was performed on SPSS (SPSS v27, IBM SPSS statistics Inc, Chicago, IL, USA) and statistical significance was set at *p* < 0.05. A mixed analysis of variance (ANOVA) was used to examine any interaction effect between gender and intervention. Primary variables (GET, $\dot{V}$O_2peak_ and peak power output) measured during the final three visits (CON, SHAM and IPC) were examined for within-group effects across condition using a one-way repeated measures ANOVA. In the event of a significant F ratio, post hoc comparisons were made using a Bonferroni correction. Where sphericity could not be assumed, a Greenhouse − Geisser correction was applied. GET expressed as a percentage of $\dot{V}$O_2peak_ was analysed using a Friedman non-parametric test with Wilcoxon post hoc tests applied in the event of a significant value. The effect size (ES) was calculated using Cohen’s d and interpreted according to the following criteria: 0.2, a small difference; 0.5, a moderate difference; 0.8 a large difference (Cohen, 1988). The data are presented as mean ± SD.

## Results

Prior to SHAM and IPC, participants completed three baseline tests which were used for the calculation of coefficients of variation for GET (CV = 4.8%, typical error = 0.08 L/min) and related variables (Table S2). To examine if effects of IPC on GET were sex-dependent, a mixed ANOVA with sex and condition as main effects was performed, revealing no significant effect of sex or interaction for GET (*F*_(1, 8)_ = 1.29, *p* = 0.29). Descriptive data for males and females are shown in Table S3. For the remaining analyses data for males and females were combined and descriptive data for key variables under control, SHAM and IPC are shown in Table 1.

**Table 1:** Test results from the cycling ramp test for CON, SHAM and IPC conditions

	CON	SHAM	IPC
GET (L O_2 _min^−1^)	1.74 ± 0.55	1.73 ± 0.56	1.89 ± 0.51*
GET (% $\dot{V}$O_2peak_)	60 ± 9	60 ± 9	65 ± 7*
GET (W)	122 ± 40	120 ± 39	133 ± 36*
VT_1_ (L O_2 _min^−1^)	1.81 ± 0.55	1.77 ± 0.58	1.87 ± 0.56
VT_2_ (L O_2 _min^−1^)	2.31 ± 0.57	2.29 ± 0.61	2.32 ± 0.59
VT_2_ (% $\dot{V}$O_2peak_)	81 ± 8	81 ± 5	81 ± 7
$\dot{V}$O_2_peak (L O_2 _min^−1^)	2.85 ± 0.70	2.84 ± 0.73	2.87 ± 0.68
Peak power (W)	214 ± 49	212 ± 51	217 ± 50 *
Peak HR (bpm)	174 ± 14	172 ± 10	174 ± 12

All data are shown as mean ± SD (*N* = 10)

*Significant difference between conditions (*P* < 0.05). See main text for definitions

GET was identified using trimmed $\dot{V}$CO_2_-$\dot{V}$O_2_ series and verified using the onsets of the abrupt changes in end-tidal FO_2_ and FCO_2_. Across all five tests (3 baseline tests, SHAM, IPC) there was no significant difference in the $\dot{V}$O_2_ between GET and abrupt changes in FO_2_ and FCO_2_. The abrupt decline in FCO_2_ was delayed with respect to the abrupt rise in FO_2_ (Fig. S1). When all estimates were combined there was a significant main effect of test (*F* = 3.86, *P* < 0.05) with higher values for IPC than other conditions (Table S4).

**Fig. 2:**
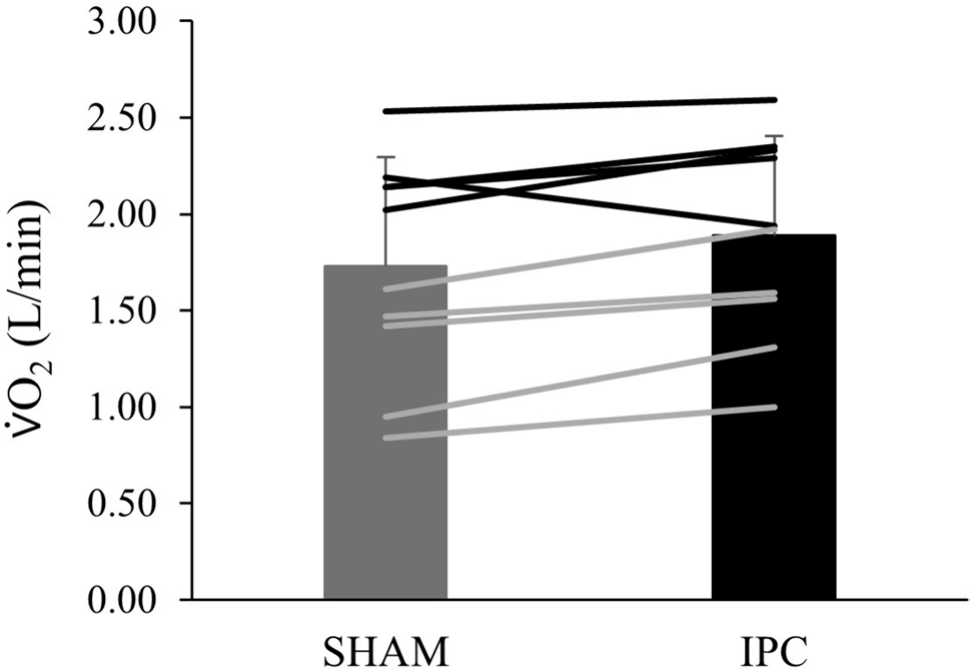
Rate of oxygen uptake ($\dot{V}$O_2_) (L/min) at the gas exchange threshold for sham and ischemic preconditioning (IPC) during the cycling ramp test. Individual (males = black lines, females = grey lines) and mean data (bars) ± SD, (*N* = 10)

In separate analysis of GET estimates based on the V-slope method, there was a significant effect of condition on GET (L O_2_^.^min^−1^) (*F*_(1.262, 11.356)_ = 5.54, *p* < 0.05) with a significant difference between IPC and CON (*p* < 0.05, ES = 0.28) but not IPC and SHAM (*p* = 0.06, ES = 0.29) (Fig. 2). For GET (%$\dot{V}$O_2peak_), there was a significant effect of condition (χ2_(2)_ = 9.80, *p* < 0.05) with a significant difference between IPC and CON (Wilcoxon *Z* = -2.50, *p* < 0.05, ES = 0.64) and IPC and SHAM (Wilcoxon *Z* = -2.25, *p* < 0.05, ES = 0.66). For GET (W), there was a significant effect of condition (*F*_(2, 18)_ = 5.42, *p* < 0.05) with a significant difference between IPC and CON (*p* < 0.05, ES = 0.30) and IPC and SHAM (*p* < 0.05, ES = 0.35).

**Fig. 3:**
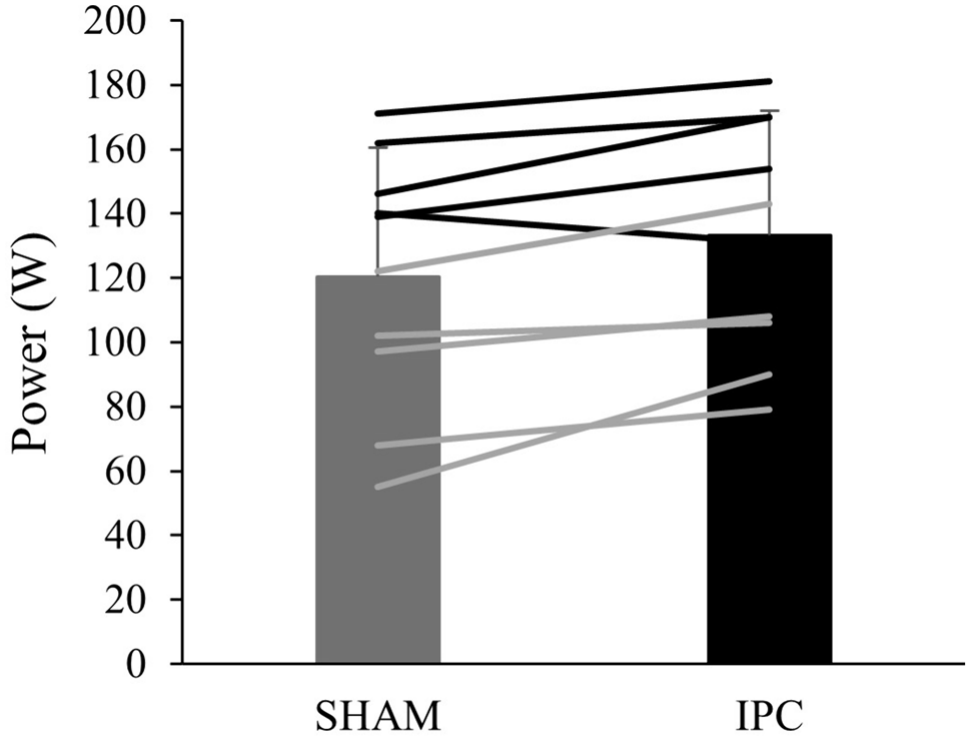
Power output at task failure for sham and ischemic preconditioning (IPC) during the cycling ramp test. Individual (males = black lines, females = grey lines) and mean data (bars) ± SD, (*N* = 10). * Significant difference between conditions (*P* < 0.05)

Unlike GET, there was no significant main effect of condition (*p* > 0.05) on VT_1_ (*F*_(1.155, 10.393)_ = 1.77), VT_2_ (*F*_(2, 18)_ = 0.55), $\dot{V}$O_2peak_ (*F*_(2, 18)_ = 0.53), $\dot{V}$O_2_ slope (*F*_(2, 18)_ = 0.25), HR slope (*F*_(2)_ = 0.16, *p* = 0.98), or peak HR (*F*_(2, 18)_ = 1.96) (Table S5). However, there was a main effect of condition on peak power output (*F*_(2, 18)_ = 4.78, *p* < 0.05) although there were no significant differences between IPC and CON (*p* = 0.22, ES = 0.05) or IPC and SHAM (*p* = 0.05, ES = 0.10) (Fig. 3).

With respect to NIRS responses, three of the 10 participants had a skinfold larger than the distance required (< 15 mm) for accurate measurements (24 ± 3 mm, *N* = 3) and were omitted from analyses. Skinfold thickness in remaining participants was 13 ± 2 mm, which is large compared with half of the inter-optode distance and thus the amount of muscle tissue actually included in the sample volume might have been small. In these participants, TSI (%) in the control condition declined by a maximum of 17 ± 9% during ramp exercise. There was a significant effect of condition on the TSI-time product measured until GET (*F*_(2, 18)_ = 5.099, *p* = 0.03) with a significant difference between CON and SHAM (*p* = 0.04) but not between IPC and CON (*p* = 0.12) or IPC and SHAM (*p* = 1.00). There was no significant effect of condition on the TSI-time product over the whole test (*F*_(2, 18)_ = 3.44, *p* = 0.07) (Table S5). Statistics related to the goodness-of-fits of data to the various functions are shown in Table 2.

**Table 2:** Statistics related to the goodness-of-fit for the primary relationships (*x*–*y*) analysed and selected primary variables (VT1, VT2, GET, $\dot{V}$O_2peak_) related to some of these relationships ($\dot{V}$O_2_-$\dot{V}$_E_, $\dot{V}$O_2_-$\dot{V}$CO_2_, time-$\dot{V}$O_2_) under the three experimental conditions (CON, SHAM, IPC)

	Statistic	CON	SHAM	IPC
$\dot{V}$O_2_-$\dot{V}$_E_ (L min^−1^)				
	*R*^2^	0.983 ± 0.024	0.986 ± 0.017	0.983 ± 0.027
	FSE	2.13 ± 0.71	1.92 ± 0.69	2.01 ± 0.64
VT1 (L O_2_ min^−1^)	95% CI	0.20 ± 0.12	0.23 ± 0.28	0.19 ± 0.10
VT2 (L O_2_ min^−1^)	95% CI	0.11 ± 0.05	0.15 ± 0.17	0.14 ± 0.10
$\dot{V}$O_2_-$\dot{V}$CO_2_ (L CO_2 _min^−1^)				
	*R*^2^	0.990 ± 0.008	0.992 ± 0.006	0.989 ± 0.007
	FSE	0.05 ± 0.02	0.04 ± 0.01	0.04 ± 0.01
GET (L O_2_ min^−1^)	95% CI	0.25 ± 0.23	0.18 ± 0.08	0.20 ± 0.16
Time-$\dot{V}$O_2_ (L O_2 _min^−1^)				
	*R*^2^	0.953 ± 0.052	0.960 ± 0.030	0.968 ± 0.020
	FSE	0.14 ± 0.08	0.14 ± 0.07	0.13 ± 0.06
$\dot{V}$O_2peak_ (L O_2_ min^−1^)	95% CI	0.53 ± 0.15	0.56 ± 0.19	0.55 ± 0.27
Time-FeO_2_				
	*R*^2^	0.931 ± 0.042	0.925 ± 0.055	0.910 ± 0.074
	FSE	0.0025 ± 0.0006	0.0024 ± 0.0006	0.0026 ± 0.0007
Time-FeCO_2_				
	*R*^2^	0.914 ± 0.075	0.901 ± 0.093	0.906 ± 0.074
	FSE	0.0014 ± 0.0003	0.0013 ± 0.0003	0.0014 ± 0.0003
Power-$\dot{V}$O_2_ (L O_2 _min^−1^)				
	*R*^2^	0.968 ± 0.014	0.968 ± 0.013	0.971 ± 0.013
	FSE	0.12 ± 0.03	0.12 ± 0.04	0.12 ± 0.04
Time-HR (beats min^−1^)				
	*R*^2^	0.995 ± 0.003	0.996 ± 0.002	0.995 ± 0.002
	FSE	1.8 ± 0.4	1.8 ± 0.6	1.9 ± 0.4
Time-TSI (%)				
	*R*^2^	0.979 ± 0.015	0.978 ± 0.013	0.988 ± 0.005
	FSE	0.5 ± 0.2	0.5 ± 0.2	0.4 ± 0.2

Mean and SD values are shown. *R*^2^unadjusted coefficient of determination, *FSE* fitted standard error and is equivalent to the standard error of the regression in same unit as *y* variable (shown in parenthesis); 95% CI = 95% confidence interval in same unit as variable

## Discussion

Effects of IPC on exercise performance and underlying mechanisms are unclear. The design of the present study was based on the possibility that any exercise-related effect of IPC is intensity-dependent and, accordingly, our experiment was designed to detect the emergence of a physiological effect of IPC across a wide range of exercise intensities within a single test. The present findings support the hypothesis that IPC increases the GET but did not support the hypothesis that IPC improves $\dot{V}$O_2peak_ or other measurements at higher intensities beyond the GET. These findings suggest that effects of IPC are evident at intensities well below peak $\dot{V}$O_2_, which should help guide further investigation of exercise-related effects of IPC.

Most studies that have tested effects of IPC on exercise performance have used fixed intensity tests (Caru et al. 2019), whereas fewer studies have used incremental exercise protocols (Bailey et al. 2012, Cheung et al. 2020, Clevidence et al. 2012, Cocking et al. 2017, Cruz et al. 2015, de Groot et al. 2010, Kaur et al. 2017, Kido et al. 2015, Kilding et al. 2018, Sabino-Carvalho et al. 2017) and only three of which have assessed measurements related to the gas exchange threshold (Bailey et al. 2012; Kilding et al. 2018; Sabino-Carvalho et al. 2017). The measurement of GET, otherwise known as the ‘anaerobic threshold’ (Beaver et al. 1986; Sietsema et al. 2021), requires the use of a maximal graded test. This test is useful to the study of IPC effects and their potential dependence on exercise intensity, given the large changes in metabolic rate and scope of physiological adjustment which occurs between rest and the metabolic limit defined by peak $\dot{V}$O_2_.

In the present study, the estimation of GET and related measurements was based on the several steps of analysis linked to the fitting of data to various functions, along with an experimental sequence of baseline tests that yielded control estimates prior to SHAM and IPC interventions. These control estimates did not differ significantly from those obtained under SHAM conditions. The coefficients of variation of these control estimates were low (Table S2) and indicative of techniques suited to detection of small physiological and performance effects of IPC. With respect to GET (L O_2_^.^min^−1^), its detection based on the $\dot{V}$CO_2−_$\dot{V}$O_2_ relationship (‘V-slope method’) yielded similar estimates to those obtained from abrupt changes in end-tidal respiratory gases (Table S4). A positive effect of IPC on GET was observed in both sexes, in all but one participant, and with an overall effect (vs SHAM) that was approximately twice the magnitude of its coefficient of variation under control conditions. In contrast, the effects of IPC on RCT and $\dot{V}$O_2peak_ were insignificant and much less than their coefficients of variation under control conditions. This is clear evidence of a targeted effect of IPC on the GET.

This finding differs from one other study which tested the effects of IPC on GET. Kilding et al. (2018) studied nine male cyclists using a continuous incremental cycling protocol and reported a lack of significant effect of IPC on the gas exchange threshold. However, there are several concerns with this study, including (1) the high estimates of gas exchange threshold (77–80%$\dot{V}$O_2peak_) which are more consistent with estimates of the RCT (Beaver et al. 1986), (2) that the entire $\dot{V}$CO_2_-$\dot{V}$O_2_ data series was included in analysis rather than a trimmed series as originally described (Beaver et al. 1986), (3) use of visual inspection for threshold detection which is known to be highly variable (Gladden et al. 1985), and (4) use of a portable gas analysis system of questionable accuracy (Macfarlane and Wong 2012; Vogler et al. 2010).

Two other studies assessed effects of IPC on blood lactate responses during discontinuous, incremental treadmill protocols and reported conflicting findings. Sabino-Carvalho et al. (2017) measured exercise blood lactate responses in 14 middle- and long-distance runners and reported no significant effect of IPC on the lactate threshold estimated with the use of curve-fitting. However, since the intensity of the lowest effort in the incremental protocol used in this study evoked a $\dot{V}$O_2_ (~ 70–75% $\dot{V}$O_2peak_) well above expected values for GET (Table 1), then it is likely that the protocol used was not optimal for lactate threshold detection (Beaver et al. 1986). In contrast, Bailey et al. (2012) tested the effect of IPC on exercise blood lactate responses in 13 young healthy men using a protocol with lower initial intensities (≈ 65%$\dot{V}$O_2peak_) and perhaps better optimised for lactate threshold detection. They observed a significant reduction in the intensity-dependent increase in blood lactate during exercise that was linked to a significant delay in achievement of a blood lactate value of 4 mmol^.^L^−1^ (i.e. ‘onset of blood lactate accumulation’). Differences in blood sampling techniques between these studies (arterialised earlobe *versus* venous catheterisation) might have also contributed to the different outcomes.

In the present study, the ~ 10% improvement in GET (W) coincided with a much smaller improvement in peak power (~ 2%) that was of marginal statistical significance, as well as a lack of effect on VT_2_ (i.e. RCT). These observations highlight a dissociation between effects on the GET and RCT and suggest a waning influence of the mechanism underlying the effect of IPC on GET as exercise progresses beyond GET towards the limit of exercise tolerance. That the effect of IPC on GET was much larger than the effect on peak power, an estimate of exercise performance, is also relevant to understanding the small and variable performance effects of IPC (~ 1–4% improvement) reported in other studies (Salvador et al. 2016). Others have observed that IPC-induced changes in muscle metabolic function do not necessarily translate to improvements in performance of exercise performed at an intensity well above GET (Peden et al. 2022). The present findings raise the hypothesis that the greatest ergogenic effect of IPC will be observed at an intensity approximating GET.

**Fig. 4:**
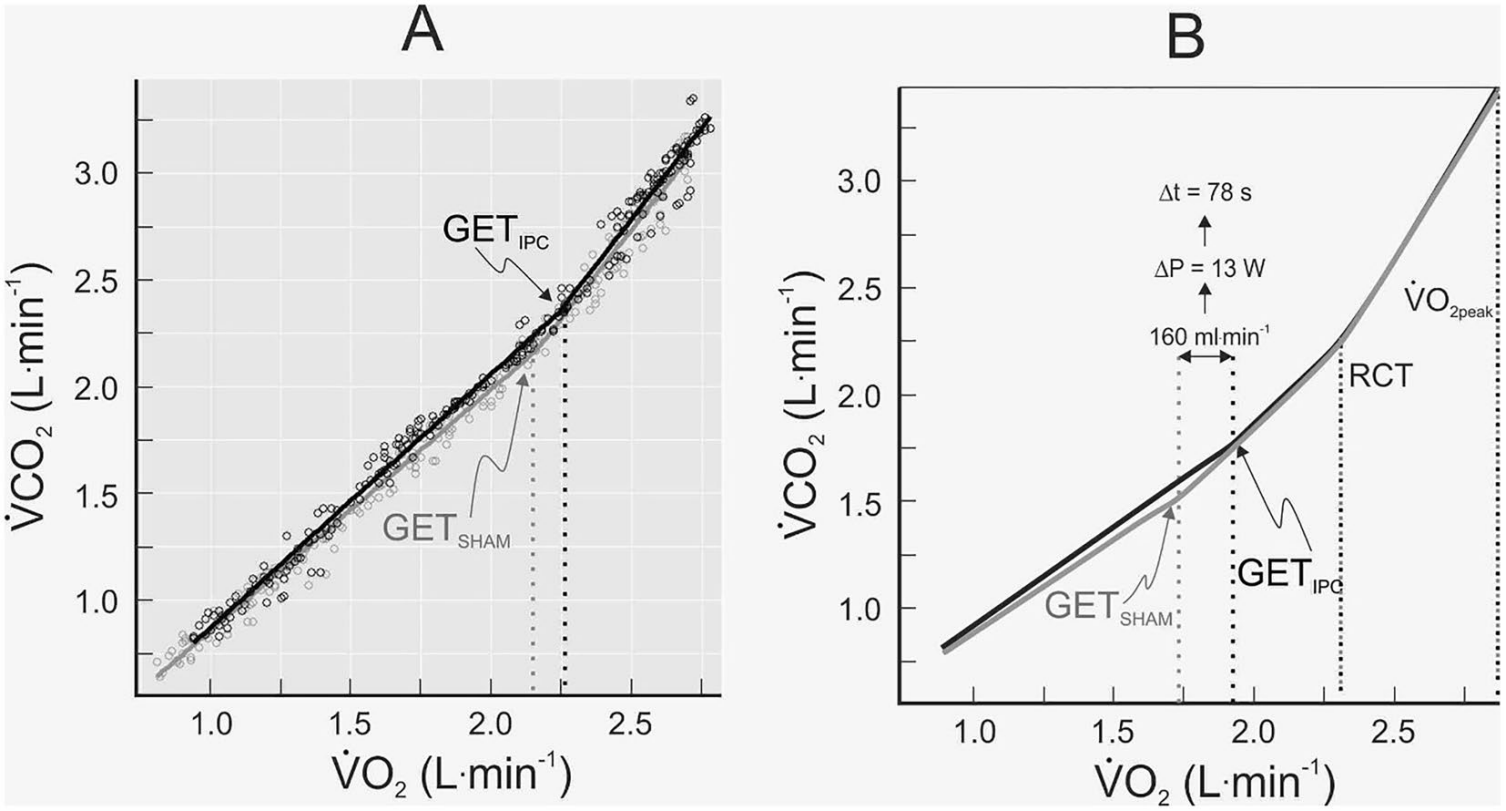
**A**: Breath-by-breath data for the rate of CO_2_ production ($\dot{V}$CO_2_) relative to rate of oxygen uptake ($\dot{V}$O_2_) during IPC and SHAM tests for a male subject. This subject was chosen because the overall effects of IPC on this subject’s responses were representative of group-averaged effects of IPC on the gas exchange threshold (GET). Lines of best fit to trimmed datasets are shown (see Methods). **B**: A cartooned illustration of the averaged effects of IPC on the $\dot{V}$CO_2_-$\dot{V}$O_2_ relationship, GET, respiratory compensation threshold (RCT) and peak $\dot{V}$O_2_ ($\dot{V}$O_2peak_) for the whole cohort (*N* = 10). The average effect of IPC on $\dot{V}$O_2_ at the GET (160 ml^.^min^−1^) was associated with an average increase in power of 13 W and a delay in the onset of the GET of 78 s

The combination of thorough familiarisation of subjects to the test protocol and GET detection based on a large number of measurements and rigorous analytical approach revealed a small ‘window of opportunity’ for the targeted effect of IPC on GET to be observed (Fig. 4A). The mean effect of IPC on the $\dot{V}$O_2_ at GET (160 ml O_2_^.^min^−1^) translated to an additional 13 W of power, which is equivalent to an economy estimate of 12.3 ml O_2_^.^min^−1.^W^−1^ and similar to other measurements of exercise economy near metabolic transition points during incremental exercise (Green and Dawson 1995; Zoladz et al. 1995). That the effect of IPC on peak power (5 W) was smaller than its effect at GET demonstrates a waning ergogenic effect of IPC as exercise progresses beyond GET towards $\dot{V}$O_2peak_. Based on the extent to which IPC delays the GET (mean delay = 78 s) during a long ramp test (Table S1), it would appear as if the window of opportunity (Fig. 4B) to observe this effect is relatively brief and might help explain the many negative findings in the literature (Caru et al. 2019; Salvador et al. 2016).

It would seem that any mechanism contributing to a delay in GET might be activated at any time prior to reaching GET. None of the present measurements made at and below GET (< 60% $\dot{V}$O_2peak_) related to metabolism ($\dot{V}$O_2_-time, $\dot{V}$CO_2_-time), muscle oxygenation (TSI-time product) or cardiorespiratory responses (HR-time, VT_1_) were affected by IPC. Others have reported that IPC does not affect $\dot{V}$O_2_, RER, HR or ventilation rate at a low intensity (~ 50% $\dot{V}$O_2peak_) (Clevidence et al. 2012). However, these measurements do not necessarily reflect the altered dynamics of CO_2_ relative to O_2_ exchange associated with increased arterial blood lactate concentration (Beaver et al. 1981). Mitochondria appear to be a prime target of IPC (Dolowy 2019). If the mechanism of the IPC effect on $\dot{V}$CO_2_ and $\dot{V}$O_2_ dynamics (GET) and blood lactate accumulation is linked to altered mitochondrial function, then factors involved in respiratory control (ATP, ADP, Pi, NADH, NAD + , Ca^++^) and which influence the balance of pyruvate production (glycolysis/glycogenolysis) and its oxidation in mitochondria are implicated. Exercise at moderate and higher intensities impairs mitochondrial function in active skeletal muscle by reducing maximal mitochondrial respiratory flux or increasing mitochondrial proton leak (Lewis et al. 2021; Trewin et al. 2018). IPC reduces mitochondrial proton leak (complex I) (Peden et al. 2022) and thus appears to offer some protection against a loss of mitochondrial function. How such protection by IPC would translate to altered $\dot{V}$CO_2_ and $\dot{V}$O_2_ dynamics during ramp exercise needs to be established.

There are several limitations with the present study. Although data for male and female participants were combined (see Results), we noted that there was a proportionally larger effect of IPC on GET in females but in association with a significantly shorter test duration (Table S3). Potential influences of sex and/or test duration on effects of IPC on GET needs further study. Pulmonary gas exchange measurements were not true breath-by-breath measurement because they were based on F_E_O_2_ and F_E_CO_2_ measurements effectively averaged over a small number of breaths (~ 2–4) that depended upon the ventilation rate. However, the time-dependent change in ventilation rate up to GET was not affected by IPC and the ‘pseudo’ breath-by-breath technique is not necessarily inferior to conventional breath-by-breath measurements which employ various methods of interpolation and smoothing. NIRS data are influenced by the thickness of subcutaneous tissue and, in the present study, might have resulted in a small sample volume and suboptimal assessment of NIRS changes in muscle tissue. Consistent with this, three participants were excluded from analysis of NIRS responses and there was a wide range in the maximum change in the exercise TSI (%) response of 8–30%. It is difficult to create a true placebo for IPC, but our use of a sham technique was combined with instructions aimed at minimising bias in participants’ expectations between SHAM and IPC. We used a heterogenous sample consisting largely of recreationally active participants and cannot extrapolate our findings to elite athletes or sedentary subjects. However, the study included a wide range of cardiorespiratory fitness levels based on $\dot{V}$O_2peak_ (Green and Askew 2018) that varied between 31–59 mL min^−1.^kg^−1^, with 90% of participants improving following IPC suggesting that it had a positive effect on the GET across a range of fitness levels.

## Conclusion

In conclusion, the present findings show that IPC improves GET without significant effect on other threshold measurements and $\dot{V}$O_2peak_. This raises the possibility that the largest ergogenic effect of IPC will be observed at intensities which approximate GET.

### Supplementary Information

Below is the link to the electronic supplementary material.

Supplementary file1 (DOCX 672 KB)

## Data Availability

The datasets generated during and/or analysed during the current study are available from the corresponding author on reasonable request. Additional data are provided as supplementary material to this manuscript.
