# Supplementary material for: Targeted effect of ischemic preconditioning on the gas exchange threshold in healthy males and females
Source: Eur J Appl Physiol. 2024 Apr 20;124(9):2697–706. doi: 10.1007/s00421-024-05481-8 (PMC11365842; doi:10.1007/s00421-024-05481-8)
Supplement: Supplementary file 1 — Supplementary file1 (DOCX 672 KB) [file 421_2024_5481_MOESM1_ESM.docx]

**
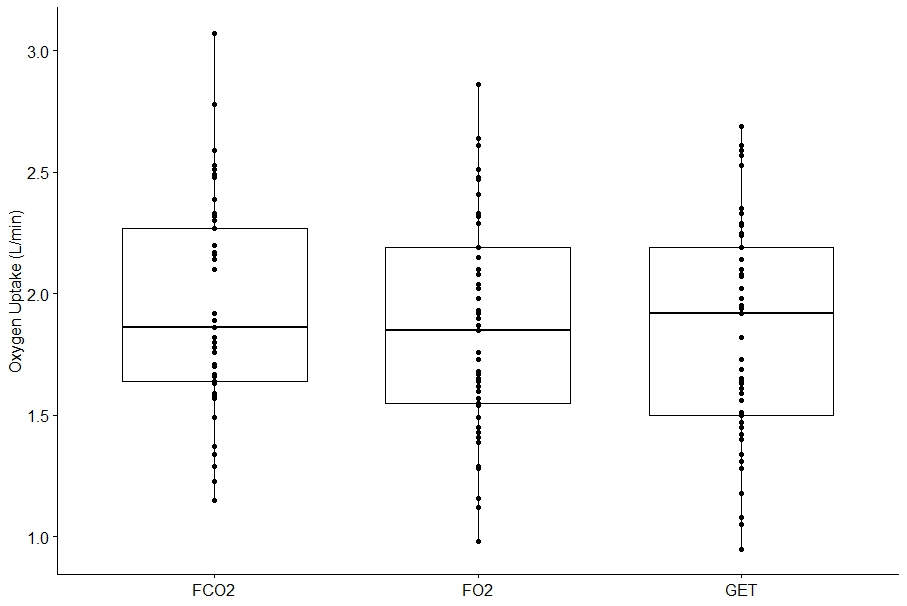
**

**Figure S1.** The rate of oxygen uptake at the three measurements used to estimate and confirm the estimation of the gas exchange threshold. Each boxplot shows measurements from all subjects under control, SHAM and IPC conditions. A two-way ANOVA revealed that there was no significant effect of measurement (FCO_2_, FO_2_, GET), test (control, SHAM, IPC) or interaction.

| **A B** |
| --- |
| 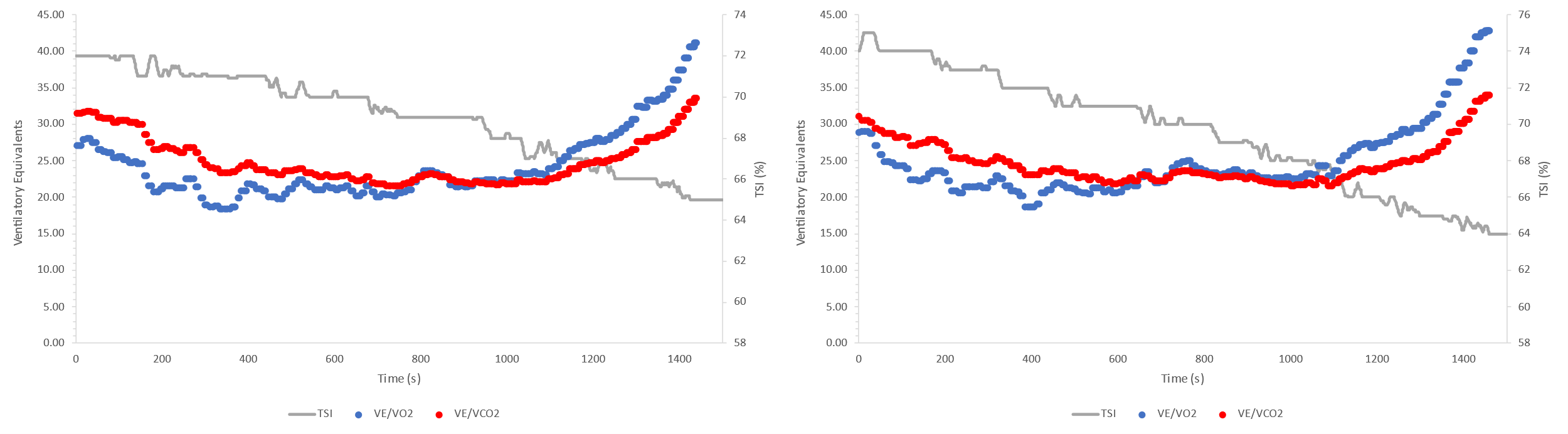 |

**Figure S2.** Representative examples of TSI responses (grey lines) from a single subject during ramp exercise in SHAM (A) and IPC (B) along with ventilatory equivalents for O_2_ (blue lines) and CO_2_ (red lines) for reference. Areas under the TSI responses (TSI-time integral) were not significantly different.

**Table S1** A description of variables and functions used to identify estimates of respiratory thresholds, slopes and peak V̇O_2_ during the cycling ramp test. Parameter estimates were unrestricted and allowed to vary from -∞ to +∞. For all functions, the conditional expressions (F1, F2 and F3) restrict the fitting of a term to the onset of the phase it is applied to and until the end of the data series.

| Estimate | Variables  (x versus y) | Function | Comment |
| --- | --- | --- | --- |
| VT_1_  VT_2_ (RCT) | V̇O_2_ vs V̇_E_ | *y* = a + b*x* + c(*x*-d)F1 + e(*x*-f)F2 + g(*x*-h)F3  F1=if(*x*<d,0,1), F2=if(*x*<f,0,1), F3=if(*x*<h,0,1) | A four-phase function. *x* = V̇O_2_ (L^.^min^-1^), *y* = V̇_E_ (L^.^min^-1^). Parameters: a = y-intercept; b = phase 1 slope; c, e and g = change in slope relative to phase 1, plus phase 2 and plus phase 3, respectively; d, f and h = thresholds between phases 1 and 2, 2 and 3, as well as 3 and 4, respectively. Parameters f and h represent VT_1_ and VT_2_. |
| GET - V̇O_2_ | V̇O_2_ vs V̇CO_2_ | Step 1: *y* = a + b*x* + c(*x*-d)F1 + e(*x*-f)F2  F1 = IF(*x*<d,0,1), F2 =if(*x*<f,0,1)  Step 2: y = a + b*x* + c(*x*-d)F1  F1=if(*x*<d,0,1) | Analysis is performed using two functions in two steps. *x* = V̇O_2_ (L^.^min^-1^), *y* = V̇CO_2_ (L^.^min^-1^). Parameters: a = y-intercept; b = phase 1 slope; c and e = change in slope relative to phase 1 and plus phase 2, respectively; d, f and h = thresholds between phases 1 and 2, 2 and 3, as well as 3 and 4. In Step 1, parameters d and f represent an ‘initial adaptation threshold’ and GET, respectively. However, this step is used to identify the initial adaptation threshold, exclude data below it, and then fit a simpler function (single threshold) in Step 2. In Step 2 parameter d represents GET. |
| GET - Power | Power vs V̇O_2_ | *y* = a + b*x* + c(*x*-d)F1 + e(*x*-f)F2 - (g exp(-(h/(*x*-i)))*x*)F3  F1=if(*x*<=d,0,1), F2=if(*x*<=f,0,1), F3=if(*x*<=i,0,1) | A four-phase function with three linear phases and a final exponential phase. *x* = power (W), *y* = V̇O_2_ (L^.^min^-1^). The power output corresponding to the V̇O_2_ at GET (from above) is estimated using this V̇O_2_-power function. Parameters: a = y-intercept; b = phase 1 slope; c and e = change in slope relative to phase 1, plus phase 2, respectively; d, f and i = thresholds between phases 1 and 2, 2 and 3, as well as 3 and 4, respectively; g = amplitude of phase 1; h = rate constant of phase 4. |
| FO_2et_-time | Time vs FO_2_  Time vs V̇O_2_ | y = a + b*x* + c(*x*-d)F1 + e(*x*-f)F2 + g(*x*-h)F3  F1=if(*x*<d,0,1), F2=if(*x*<f,0,1), F3=if(*x*<h,0,1) | A four-phase function. *x* = time (s), *y* = FO_2_. Parameters: a = y-intercept; b = phase 1 slope; c, e and g = change in slope relative to phase 1, plus phase 2, plus phase 3, respectively; d, f and h = time delays (thresholds) of phases 2, 3 and 4, respectively. Parameter f is used to represent the onset of a sustained increase in FO_2_ and is indicative of GET. The V̇O_2_ at this time is identified using the V̇O_2_-time relationship (see below). |
| FCO_2et_-time | Time vs FCO_2_  Time vs V̇O_2_ | y = a + b*x* + c(*x*-d)F1 + e(*x*-f)F2 + g(*x*-h)F3  F1=if(*x*<d,0,1), F2=if(*x*<f,0,1), F3=if(*x*<h,0,1) | A four-phase function. *x* = time (s), *y* = FCO_2_. Parameters: a = y-intercept; b = phase 1 slope; c, e and g = change in slope relative to phase 1, plus phase 2, plus phase 3, respectively; d, f and h = time delays (thresholds) of phases 2, 3 and 4, respectively. Parameter f is used to represent the onset of a sustained decrease in FCO_2_ and, in conjunction with the above-mentioned FO_2_ threshold, is indicative of GET. The V̇O_2_ at this time is identified using the V̇O_2_-time relationship (see below). |
| V̇O_2peak_  V̇O_2_ slope | Time vs V̇O_2_ | y = a + b*x* + c(*x*-d)F1 + e(*x*-f)F2 - (g exp(-(h/(*x*-i)))*x*)F3  F1=if(*x*<=d,0,1), F2=if(*x*<=f,0,1), F3=if(*x*<=i,0,1) | A four-phase function with three linear phases and a final exponential phase. *x* = time (s), *y* = V̇O_2_ (L^.^min^-1^). Parameters: a = y-intercept; b = phase 1 slope; c and e = change in slope relative to phase 1, plus phase 2, respectively; d, f and i = time delays of phases 2, 3 and 4, respectively; g = amplitude of phase 1; h = time constant of phase 4. The V̇O_2peak_ corresponds to the maximum value obtained using this function. The V̇O_2_ slope is represented by the V̇O_2_-time relationship up to the approximate time at which GET occurs (i.e. sum of parameters b and c). |
| HR slope | Time vs HR | y = a + bx + c(x-d)F1 + e(x-f)F2  F1=if(x<=d,0,1), F2=if(x<=f,0,1) | A three-phase function. *x* = time (s), *y* = heart rate (beats^.^min^-1^). Parameters: a = y-intercept; b = phase 1 slope; c and e = change in slope relative to phase 1, as well as plus phase 2, respectively; d and f = thresholds between phases 1 and 2, as well as 2 and 3, respectively. The slope of HR-time relationship for the entire test is the sum of parameters b, c and e. |
| TSI-time | Time vs TSI | y = a + b(1-exp(-(x/c))) + d(x-e)F1 + f(x-g)F2 + h(exp((x-i)/j)-1)F3  F1=if(x<e,0,1), F2=if(x<g,0,1), F3=if(x<i,0,1) | A four-phase function with two linear and two exponential phases. Parameters: a = y-intercept; b = amplitude of phase 1; c = time constant of phase 1; d and f = slopes of phases 2 and 3, respectively; e, g and I = time delays for phases 2, 3 and 4, respectively; h = amplitude of phase 4; j = time constant of phase 4. Integration of this function over two periods of time (up to GET and total test time) yields estimates of areas under the TSI-time response over these periods (unit = % s). |

FCO_2et_, end-tidal fraction of carbon dioxide; FO_2et,_ end-tidal fraction of oxygen; GET, gas exchange threshold; HR, heart rate; RCT, respiratory compensation threshold; TSI, tissue saturation index; V̇_E_, rate of expired ventilation; V̇CO_2_, rate of carbon dioxide output; V̇O_2_, rate of oxygen uptake.

**Table S2.** Coefficient of variation (CV) and typical error (TE) values for the cycling ramp test based on the first three baseline tests (i.e. not including SHAM and IPC). TE values are reported in the unit of measurement shown in the first column. Estimates of GET are based on the V̇CO_2_-V̇O_2_ relationship (Table S1).

|  | **CV** (%) | **TE** |
| --- | --- | --- |
| **GET** (L O_2_^.^min^-1^) | 4.8 | 0.08 |
| **VT_1_** (L O_2_^.^min^-1^) | 11.1 | 0.14 |
| **VT_2_** (L O_2_^.^min^-1^) | 5.2 | 0.12 |
| **V̇O_2_peak** (L.min^-1^) | 3.4 | 0.10 |
| **Peak Power** (W) | 2.2 | 4.7 |

GET, gas exchange threshold; V̇O_2_peak, peak oxygen uptake;

VT_1_, first ventilatory threshold; VT_2_, second ventilatory threshold.

**Table S3.** Estimates of selected exercise variables for males and females for CON, SHAM and IPC conditions. All data are shown as mean ± SD, (*n* = 10).

|  | **CON** | **Males**  **SHAM** | **IPC** | **CON** | **Females**  **SHAM** | **IPC** |
| --- | --- | --- | --- | --- | --- | --- |
| GET (L O_2_^.^min^-1^) | 2.18 ± 0.32 | 2.20 ± 0.19 | 2.30 ± 0.23 | 1.31 ± 0.33 | 1.26 ± 0.34 | 1.48 ± 0.34 |
| GET (% V̇O_2peak_) | 65 ± 7 | 66 ± 6 | 69 ± 6 | 55 ± 8 | 54 ± 8 | 62 ± 6 |
| GET (W) | 153 ± 25 | 152 ± 14 | 161 ± 19 | 90 ± 23 | 89 ± 27 | 105 ± 24 |
| VT_2_ (% V̇O_2peak_) | 84 ± 7 | 84 ± 5 | 84 ± 6 | 78 ± 7 | 77 ± 4 | 77 ± 6 |
| V̇O_2peak_ (L O_2_.min^-1^) | 3.35 ± 0.55 | 3.38 ± 0.55 | 3.36 ± 0.52 | 2.36 ± 0.44 | 2.29 ± 0.39 | 2.38 ± 0.43 |
| Peak Power (W) | 248 ± 40 | 248 ± 43 | 252 ± 40 | 181 ± 32 | 176 ± 28 | 182 ± 31 |

CON, control; GET, gas exchange threshold; IPC, ischemic preconditioning; VT_2_, second ventilatory threshold, equivalent to the respiratory compensation threshold; V̇O_2_peak, peak oxygen uptake.

**Table S4.** Three estimates (mean ± SD) of the gas exchange threshold (L O_2_^.^min^-1^) during five tests in nine of the ten participants. One participant was excluded because end-tidal gas behaviour could not be reliably fitted. The estimates are represented by parameters in three functions described in Table S1.

| **Estimate** | **Baseline 1** | **Baseline 2** | **CON** | **SHAM** | **IPC** |
| --- | --- | --- | --- | --- | --- |
| parameter d  GET-V̇O_2_ | 1.88 ± 0.50 | 1.73 ± 0.44 | 1.83 ± 0.50 | 1.83 ± 0.50 | 1.99 ± 0.43 |
| parameter f  FO_2et_-time | 1.87 ± 0.56 | 1.80 ± 0.36 | 1.86 ± 0.57 | 1.77 ± 0.42 | 2.02 ± 0.36 |
| Parameter f  FCO_2et_-time | 2.05 ± 0.58 | 1.84 ± 0.33 | 1.94 ± 0.54 | 1.88 ± 0.34 | 2.03 ± 0.39 |

**Table S5.** Test values for the TSI-time integral below GET, TSI-time integral for the whole data series, V̇O_2_ slope & HR slope. Further information about these variables can be found in Table S1. All data are shown as mean ± SD, (TSI, *n* = 7; V̇O_2_ and HR slope, *n* = 10).

|  | **CON** | **SHAM** | **IPC** |
| --- | --- | --- | --- |
| **TSItime ≤ GET**  (% s) | 49311 ± 12958 | 51699 ± 14239 | 51642 ± 13841 |
| **TSItime total**  (% s) | 76868 ± 16541 | 80590 ± 20190 | 81966 ± 18961 |
| **V̇O_2_ slope** (mL/min/min) | 121 ± 14 | 121 ± 18 | 124 ± 11 |
| **HR slope** (beats/min/min) | 5.43 ± 1.53 | 5.43 ± 1.52 | 5.49 ± 1.74 |

CON, control; GET, gas exchange threshold; IPC, ischemic preconditioning; TSI, tissue saturation index; V̇O_2_ slope, oxygen uptake slope.
